# Impaired PIEZO1 function in patients with a novel autosomal recessive congenital lymphatic dysplasia

**DOI:** 10.1038/ncomms9329

**Published:** 2015-09-21

**Authors:** Viktor Lukacs, Jayanti Mathur, Rong Mao, Pinar Bayrak-Toydemir, Melinda Procter, Stuart M. Cahalan, Helen J. Kim, Michael Bandell, Nicola Longo, Ronald W. Day, David A. Stevenson, Ardem Patapoutian, Bryan L. Krock

**Affiliations:** 1 Howard Hughes Medical Institute, Molecular and Cellular Neuroscience, Dorris Neuroscience Center, The Scripps Research Institute, La Jolla, California 92037, USA; 2 Genomics Institute of the Novartis Research Foundation, San Diego, California 92121, USA; 3 ARUP Institute for Clinical and Experimental Pathology, ARUP Laboratories, Salt Lake City, Utah 84108, USA; 4 Department of Pathology, University of Utah, Salt Lake City, Utah 84112, USA; 5 Integrative Structural and Computational Biology, The Scripps Research Institute, La Jolla, California 92037, USA; 6 Department of Pediatrics, Division of Medical Genetics, University of Utah, Salt Lake City, Utah 84112, USA; 7 Department of Pediatrics, Division of Pediatric Cardiology, University of Utah, Salt Lake City, Utah 84112, USA; 8 Department of Pediatrics, Division of Medical Genetics, Stanford University, Stanford, California 94305, USA; 9 Department of Pathology and Laboratory Medicine, Division of Genomic Diagnostics, The Children’s Hospital of Philadelphia, Philadelphia, Pennsylvania 19104, USA; 10 Department of Pathology and Laboratory Medicine, Perelman School of Medicine, University of Pennsylvania, Philadelphia, Pennsylvania 19104, USA

## Abstract

Piezo1 ion channels are mediators of mechanotransduction in several cell types including the vascular endothelium, renal tubular cells and erythrocytes. Gain-of-function mutations in *PIEZO1* cause an autosomal dominant haemolytic anaemia in humans called dehydrated hereditary stomatocytosis. However, the phenotypic consequence of *PIEZO1* loss of function in humans has not previously been documented. Here we discover a novel role of this channel in the lymphatic system. Through whole-exome sequencing, we identify biallelic mutations in *PIEZO1* (a splicing variant leading to early truncation and a non-synonymous missense variant) in a pair of siblings affected with persistent lymphoedema caused by congenital lymphatic dysplasia. Analysis of patients’ erythrocytes as well as studies in a heterologous system reveal greatly attenuated PIEZO1 function in affected alleles. Our results delineate a novel clinical category of PIEZO1-associated hereditary lymphoedema.

## Abstract (web summary)

Lukacs *et al.* identify mutations in the *PIEZO1* gene in patients with congenital lymphatic dysplasia. The study also characterizes the functional consequence of the disease-associated Piezo1 mutant proteins and show attenuated ion channel function in cellular context.

## Graphical abstract

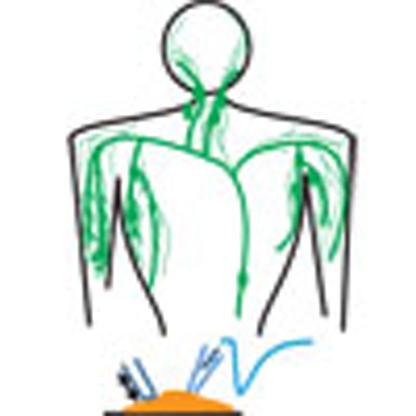

The lymphatic system is a network of thin-walled vessels important for extracellular fluid homeostasis. As an important regulator of tissue turgor, development of this network is regulated by interstitial pressure^1^. Lymphatic endothelial cells are also sensitive to shear forces induced by fluid flow, influencing lymphatic valve formation and thereby the function of conducting lymphatic vessels^2^. However, the molecular mechanism of mechanosensation in these cells is largely unknown.

Piezo1 and Piezo2 form a unique class of non-selective cation channels activated by physical stimuli, and are among the few ion channels reliably demonstrated to convey mechanical cues of the environment to mammalian cells^3^,^4^,^5^,^6^,^7^. Piezo1 plays an important physiological role in the cardiovascular, renal and haematopoietic systems, while Piezo2 is the principal sensor of mechanical force relevant for touch sensation^5^,^6^,^8^,^9^,^10^,^11^. In red blood cells, Piezo1 regulates cell volume homeostasis. Gain-of-function *PIEZO1* mutations are linked to cases of dehydrated hereditary stomatocytosis (DHS), a condition of decreased intracellular erythrocyte volume and consequent mild haemolysis^12^,^13^,^14^. Conversely, blood cell-specific *Piezo1* knockout mice exhibit increased erythrocyte size and osmotic fragility^15^. Piezo1 also plays an important role in endothelial shear stress sensing. Systemic loss of Piezo1 function in mice leads to a diminished shear-induced alignment of endothelial cells and a severe impairment of vascular development, leading to embryonic lethality^8^,^16^. The full extent to which Piezo1 channels regulate mammalian development and physiology is yet to be elucidated.

In the present study, we describe a novel role for PIEZO1 in the lymphatic system. High-throughput sequencing of a family with a congenital lymphatic dysplasia identified compound heterozygosity for a splicing and missense variant in *PIEZO1*. Functional analysis of the affected individuals’ red blood cells revealed severely diminished PIEZO1 responses to both mechanical stimuli and the recently characterized pharmacological PIEZO1-activating compound Yoda1 (ref. 17). Patch-clamp analysis of the missense PIEZO1 channel in a heterologous system (a mammalian cell line derived from HEK293T cells) indicated strongly diminished current amplitudes as compared with wild type, likely due to reduced cell surface expression. These findings suggest a role for PIEZO1 in human lymphatic development and implicate it as a novel genetic cause of an autosomal recessive congenital lymphatic dysplasia.

## Results

### Identification of PIEZO1 variants in congenital lymphoedema patients

**Figure 1:**
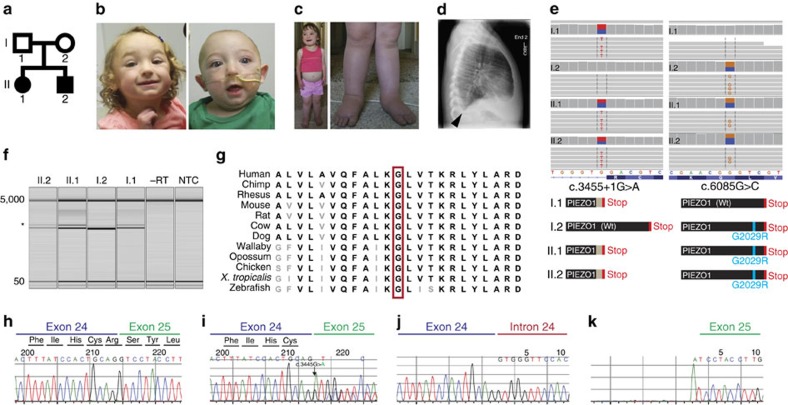
PIEZO1 variants in a novel form of hereditary lymphoedema. (**a**) Pedigree of the family described in this study. (**b**) Photographs of II.1 and II.2 at 32 and 7 months of age, respectively. Distinctive facial features include mild infraorbital hypoplasia with subjective hypertelorism, slightly flat facial gestalt with mild periorbital oedema. (**c**) Photographs of II.1 at 42 months of age. Note abdominal oedema and swelling on the apical surface of the feet. (**d**) Lateral chest X-ray of II.1 at age 42 months demonstrating pleural effusions. Blunting of the costophrenic angle, a hallmark of pleural effusions, is indicated by an arrow. (**e**) Alignment of exome sequencing reads (horizontal grey bars) from all individuals to the reference human genome illustrates both the c.3455+1G>A and c.6085G>C mutations. Vertical grey bars next to I.1, I.2, II.1 and II.2 indicate sequencing depth at each base, ∼70 × ; variant bases are highlighted in both the sequencing reads and depth of sequencing bars. The reference sequence and translation are indicated at the bottom of the image. Note that *PIEZO1* is encoded by the reverse strand, so variant calls depicted in the sequencing reads are the complement. Predicted protein products for individual family members are depicted below each column of sequencing reads. (**f**) RT–PCR analysis of patient samples shows defective splicing in those harbouring the c.3455+1G>A mutation. Asterisk indicates shifted product, which is absent in I.2. 5,000 and 50 indicate the internal size standard. (**g**) Protein alignment of a portion of the C terminus of PIEZO1 illustrating the highly conserved nature of the glycine residue at position 2,029. (**h**) Sanger sequencing results of RT–PCR products from I.2. (**i**) Sanger sequencing results of RT–PCR products from I.1. Note the double sequence starting at the exon/intron boundary indicative of retention of intron 24. (**j**) Deconvolution of dual sequences reveals retention of intron 24 in one allele, with normal splicing from the other allele. (**k**) Note the software algorithm incorrectly attributes the c.3455+1G>A variant to the exon 25 sequence, so the correct first nucleotide of intron 24 in **j** is an adenine, while the correct first nucleotide in **k** is a guanine.

A family (Fig. 1a) with a pair of sibs affected with a congenital lymphatic dysplasia was evaluated. II.1 presented at birth with hydrops and bilateral chylothorax, confirmed by fluid analysis. Her neonatal condition was severe, requiring prolonged intensive care including extracorporeal membrane oxygenation. At 42 months of age, she displays significant persistent lymphoedema of her legs, torso and face (Fig. 1b,c), chronic pleural effusions (Fig. 1d), but normal intellectual function. II.2 had minor lymphoedema at birth with swelling of the legs and scrotum. He developed pleural effusions leading to tachypnoea at 3 months of age. Overall, he had a milder clinical course than II.1, but was presumed to have the same genetic condition. Both patients and parents had blood counts including mean corpuscular volume in the normal range. On the basis of these findings, we carried out whole-exome sequencing on the parents as well as the affected sibs. Known genetic causes of congenital lymphoedema were queried, revealing no rare or pathogenic variants. On the basis of the recurrent phenotype in this family, the data were next analysed using a model for autosomal recessive inheritance. Using a population frequency cutoff of <0.1% minor allele frequency based on the 1000 Genomes project, the only candidate gene that fit this mode of inheritance was *PIEZO1*.

Both affected sibs were compound heterozygotes for a splicing variant, c.3455+1G>A and a missense variant, c.6085G>C;p.Gly2029Arg (Fig. 1e). Neither identified variant has been listed in the publicly available human genomics databases 1000 Genomes, Exome Sequencing Project or the Exome Aggregation Consortium. The c.3455+1G>A variant was inherited from I.1 and the c.6085G>C variant from I.2 (Fig. 1e). c.3455+1G>A alters the canonical splice donor motif of exon 24, and is predicted to result in the inclusion of the 80-base-pair (bp) intron 24 in the mature messenger RNA. Reverse transcription (RT)–PCR and consequent Sanger sequencing of the product confirmed insertion of this intron in all individuals in the family harbouring the c.3455+1G>A variant (Fig. 1f,h–k). This transcript is predicted to encode the peptide p.Ser1153Trpfs*21, a severely truncated product that lacks more than half the protein at the C-terminal end, including the channel pore^18^. The c.6085G>C;p.Gly2029Arg variant alters a highly conserved residue in the PIEZO1 protein (Fig. 1g), which lies immediately adjacent to a predicted transmembrane domain on the presumptive cytoplasmic side in the C-terminal part of the protein^18^. The computational programs SIFT^19^ and PolyPhen2 (ref. 20) predict this alteration to be pathogenic. These data, taken together with the involvement of this channel in vascular development, make *PIEZO1* a compelling candidate gene for a congenital lymphatic dysplasia.

### Strongly decreased function of the identified PIEZO1 variants

**Figure 2:**
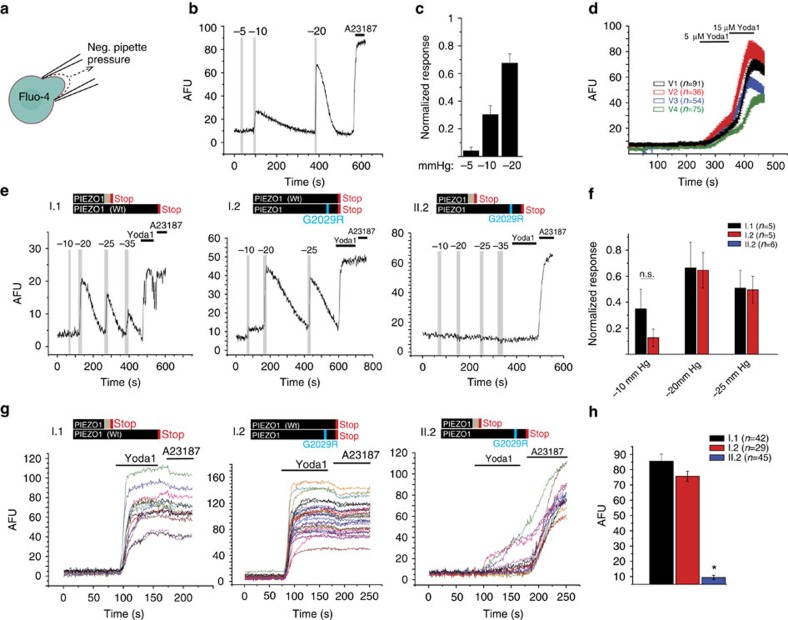
Erythrocytes of patient II.1 display severely decreased PIEZO1 function. Erythrocytes loaded with Fluo-4 were subjected to mechanical or chemical PIEZO1 stimulation. (**a**) Schematic of experimental set-up for mechanical stimulation of erythrocytes via negative pipette pressure. (**b**) Representative response of a control (healthy volunteer) red blood cell to mechanical stimulation, indicated by shaded areas on the trace. Magnitude of negative pressure is indicated above the shaded sections in mm Hg. (**c**) Average±s.e.m. of responses to negative pipette pressures in four different healthy volunteer individuals are shown as normalized to the response to the calcium ionophore A23187 (4 μM). (**d**) Average calcium responses of erythrocytes from the four volunteer individuals (depicted in different colours) to the PIEZO1 activator Yoda1 (5 and 15 μM). Cells were stimulated with the compound via whole-chamber perfusion after allowing them to settle and loosely attach to the bottom of the chamber. (**e**) Red blood cells from I.1, I.2 and II.2 were stimulated mechanically with negative pipette pressures where indicated by shaded grey bars. Numbers above grey bars denote pressure applied in mm Hg. Yoda1 and the calcium ionophore A23187 were applied via whole-chamber perfusion at the end of each measurement. (**f**) Average±s.e.m. of individual responses normalized to the calcium ionophore A23187 (4 μM). While every parental red blood cell responded to a stimulation greater than −10 mm Hg, no responses were observed to pressures as high as −35 mm Hg in the cells of patient II.2. Statistical comparison of I.1 and I.2 (*n*=5 in both groups) was performed using the Student’s *t*-test and were found to be not significantly different at the 0.05 level, *P*=0.21. (**g**) Red blood cells were stimulated with Yoda1 (15 μM) and the calcium ionophore A23187 (4 μM) as described in **d**. Background-subtracted Fluo-4 fluorescence intensities from individual cells from I.1, I.2 and II.2 are shown. (**h**) Average magnitude of calcium responses to 15 μM Yoda1 from **g** is shown for *n*=42, 29 and 45 cells of I.1, I.2 and II.2, respectively. One-way analysis was used for statistical comparison. II.2 responses were significantly different from I.1 and I.2, with *P* values of 2.53E−31 and 1.59E−23, respectively (Bonferroni means comparison test). AFU: arbitrary fluorescence unit.

We tested whether PIEZO1 function was altered in the affected sibs. It was recently shown that Piezo1 is expressed in murine erythrocytes and that negative pressure pulses applied to the membrane via a tapered-tip glass pipette resulted in calcium influx, that was absent following deletion of Piezo1 (ref. 15). To validate this assay in human erythrocytes, we obtained blood from I.1 and I.2 as well as four additional, unrelated healthy volunteers. Both parental and volunteer erythrocytes exhibited robust calcium entry upon stimulation with negative pressure (Fig. 2a–c,e,f). With the same glass pipettes used for control measurements, pressure-induced calcium responses were not observed in the erythrocytes obtained from II.2 (Fig. 2e,f). Similarly, the recently described Piezo1-activating compound Yoda1 elicited robust calcium entry in control (Fig. 2d,g,h) but not II.1 erythrocytes where it induced only minor calcium responses in a small fraction of the cells (Fig. 2g,h). These results suggest severely decreased PIEZO1 function in the affected individuals.

**Figure 3:**
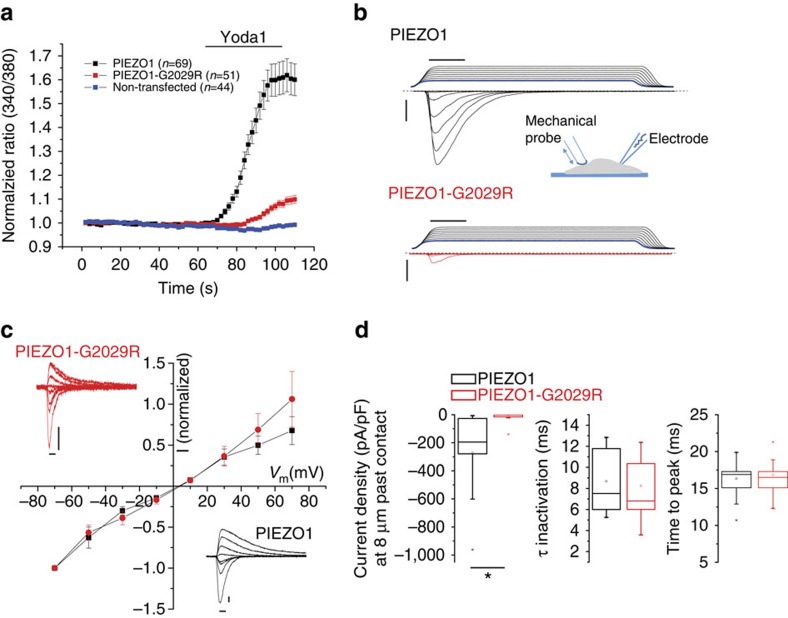
Attenuated maximal current responses in heterologous cells expressing PIEZO1-G2029R. Wild-type hPIEZO1 and hPIEZO1-G2029R were expressed in HEK-P1KO cells. (**a**) Fluorescent calcium imaging of cells loaded with Fura-2 expressing PIEZO1 or PIEZO1-G2029R constructs, both containing EGFP driven by an Internal Ribosomal Entry Site for transfection control. Average fluorescence ratios (340 nm/380 nm) of GFP-positive cells are plotted, normalized to baseline ratio values. (**b**) Representative whole-cell patch-clamp recordings performed using a blunt-ended mechanical probe, which was moved at 1-μm increments to elicit mechanically induced PIEZO1 and PIEZO1-G2029R current responses. A schematic of the measurement configuration is depicted in the inset. Dashed lines indicate zero current levels in the individual traces. A recording of the displacement steps of the mechanical probe is shown above each trace, with the first step that induced visible contact between the probe and the cell depicted in blue colour. Horizontal scale bars, 25 ms, vertical scale bars, 1 nA. (**c**) Current–voltage relationships recorded at the 3rd mechano-elicited response show no differences between wild-type and G2029R channels (*n*=5). Insets show representative recordings for wild-type (black) and G2029R (red) channels. Reversal potential was 3.4±1.08 mV and 3.83±0.45 mV for wild-type and G2029R channels, respectively. Horizontal scale bars, 10 ms, vertical scale bars, 0.1 nA (**d**) Analysis of mechanically induced current traces shows strongly decreased maximal current densities in the PIEZO1-G2029R channel. Statistics are shown for the mechano-stimulation step 8 μm past where the probe made visible contact (left). Analysis of inactivation and activation kinetics (centre and right, respectively) of the 3rd mechanically induced response (that is, 2 μm past the mechanical threshold) revealed no differences. *n*=9 and 12 for wild-type and G2029R channels, respectively. Whiskers in the boxplot depict 1.5 interquartile range, star marks indicate range. Statistical analysis was performed using the Mann–Whitney *U*-test. At the 0.05 level, only current densities were significantly different (*P*=0.0023).

As erythrocytes from both I.1 and I.2 exhibited relatively normal PIEZO1 responses, we performed a more in-depth analysis of the p.G2029R variant. To enable reliable comparison between the function of this variant and the wild-type PIEZO1 channels, we generated a HEK293T-derived cell line lacking endogenous PIEZO1 (HEK-P1KO, see Methods). Similar to the red blood cells of II.2, HEK-P1KO cells expressing the PIEZO1-G2029R channel displayed a strongly diminished calcium response to Yoda1 as compared with wild type (Fig. 3a). To assess the mechanical responsiveness of PIEZO1-G2029R, we performed whole-cell patch-clamp experiments and stimulated these cells with a blunt glass probe as depicted in the inset in Fig. 3b. The PIEZO1-G2029R channel currents displayed similar current morphology as wild-type channels (Fig. 3b–d), but with greatly decreased average current density (Fig. 3d). While the current responses were strongly diminished in most cells, some cells exhibited currents comparable in size to wild type. These results suggested that the p.G2029R mutation may alter PIEZO1 surface expression. The c.3455+1G>A variant encodes a severely truncated PIEZO1 protein, which lacks the region shown to contain the pore, and is therefore not expected to be functional.

### Missense PIEZO1 variant shows attenuated surface expression *in vitro*

**Figure 4:**
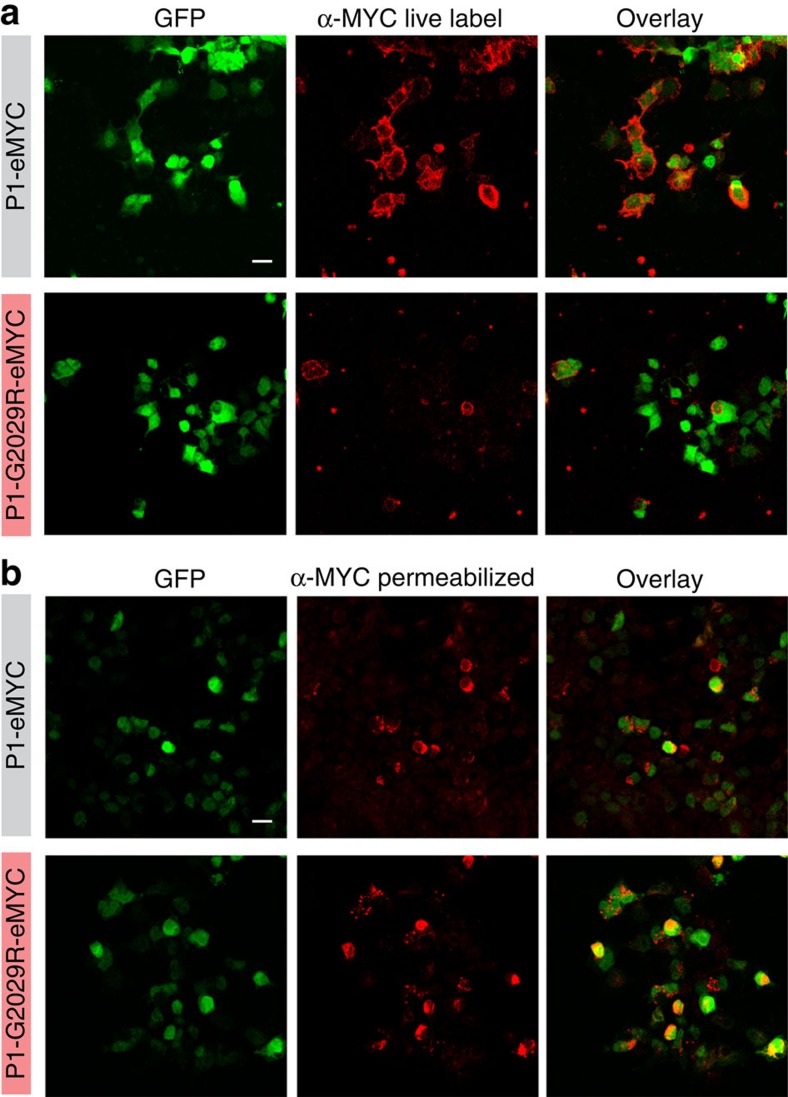
PIEZO1-G2029R shows decreased surface expression. HEK-P1KO cells were transfected with either wild-type P1-eMYC or P1-G2029R-eMYC channels. Both constructs contained and extracellular myc tag and an IRES-EGFP for transfection control. (**a**) Live, unpermeabilized immunofluorescent labelling using an anti-myc antibody show clear surface labelling of 81.5% of the GFP-positive cells in cells expressing P1-eMYC and 17.2% in P1-G2029R-eMYC. (**b**) Fixed, permeabilized cells were subjected to immunofluorescent labelling with anti-myc and show similar extent of labelling in both wild-type and G2029R groups. Scale bars, 10 μm.

To test the surface expression of PIEZO1 p.G2029R, we inserted a myc tag at amino-acid position 1,764 in both the wild-type and p.G2029R PIEZO1 channels (P1-eMYC and P1-G2029R-eMYC, respectively). This location is in a predicted extracellular loop in mouse Piezo1, and insertion of a myc tag at this location did not alter channel function^18^. Immunofluorescent labelling of live, unpermeabilized HEK-P1KO cells expressing the P1-eMYC channel confirmed surface expression of this construct in transfected cells, as 81.5% of transfected cells were labelled with the myc antibody. In the same assay, P1-G2029R-eMYC channels showed decreased cell surface labelling with only 17.2% of transfected cells showing discernible membrane labelling (Fig. 4a). To test whether this was due to a decreased overall expression of the PIEZO1-G2029R channel, we performed immunofluorescent myc-labelling experiments on fixed and permeabilized cells expressing the same constructs. We found similar fluorescence intensity and percentage of myc-labelled transfected cells in the p.G2029R and wild-type groups, suggesting that both constructs are expressed at comparable levels (Fig. 4b). The reduced surface labelling of cells expressing the P1-G2029R-eMYC channel is in agreement with both our patch-clamp and calcium imaging results, and together suggest that decreased function of PIEZO1-G2029R is, at least to a large extent, due to reduced channel abundance in the plasma membrane.

## Discussion

Our data implicate PIEZO1 loss of function as a novel genetic cause of an autosomal recessive congenital lymphatic dysplasia. The affected individuals have a generalized lymphatic dysplasia with some phenotypic overlap with Hennekam syndrome, an autosomal recessive generalized lymphatic dysplasia. However, Hennekam syndrome generally exhibits much more severe lymphoedema, intestinal lymphangiectasia, more severe facial dysmorphisms and intellectual disability^21^. Mild cases of CCBE1-associated Hennekam syndrome have been documented, but these individuals invariably have intestinal lymphangiectasias^21^,^22^, which was not observed in the sibs reported herein. The absence of lymphoedema in both parents, the congenital onset and persistent nature of the affected individuals’ lymphoedema and the absence of additional clinical features such as distichiasis, microcephaly, chorioretinopathy, intellectual disability, hypotrichosis, telangiectasia and lack of mutations in all genes previously associated with other known forms of inherited primary lymphoedema strongly suggest that the symptoms are part of a novel clinical entity termed PIEZO1-associated hereditary lymphoedema.

The affected individuals described are compound heterozygotes for a splicing variant and a single amino-acid substitution. c.3455+1G>A abolishes a splice donor motif, leading to a frame-shift insertion and a truncated PIEZO1 product that lacks the highly conserved C-terminal region that contains the channel pore^18^. The other allele contains a single amino-acid change from Gly to Arg at position 2,029. According to recently proposed transmembrane topologies^18^, this residue is found on a predicted intracellular loop within the aforementioned highly conserved C terminus. It is unlikely that this mutation is directly associated with the ion permeation path, as mutations of acidic residues very closely flanking this location in mouse Piezo1 had no effect on pore properties^18^. PIEZO1-G2029R channel currents were similar to those in wild type but smaller in maximal amplitude. In rare cases, the amplitude was also comparable to wild type. The residual functional variant in the affected sibs may therefore be a potential candidate for targeted phenotype rescue via chemical chaperonins.

Interestingly, a mutation of the nearby residue p.A2020T has the opposite effect; it decreases channel inactivation and is associated with DHS^12^. Some cases of DHS have been associated with perinatal oedema, which is resolved within a few months after birth^23^,^24^. While the lymphoedema persists in the patients described in the present study, the phenotypic similarity raises interesting questions. Piezo1 knockdown in mice is known to perturb alignment of vascular endothelium and therefore inhibit proper blood vessel formation^16^. It is possible that both increased and decreased sensitivity of lymphatic endothelial cells could result in similar defects in lymphatic vessel development. It is also possible, however, that the mechanism of lymphoedema development in these two cases is unrelated.

We reason that the strongly decreased surface expression in the heterologous system (Fig. 4a) provides an attractive possible molecular mechanism for the loss of PIEZO1 function in II.2 as compared with I.1, who carries one fully functional allele (Fig. 1d,e; Fig. 2a). Decreased surface abundance due to missense mutations has been documented in other channelopathies^25^,^26^. However, channel trafficking and regulation may be very different *in vivo* as compared with the heterologous cells, and may include tissue-specific variability. We therefore cannot rule out other possible mechanisms of action for this mutation. Similarly, we cannot exclude the possibility of alternative splicing resulting in a functional product from the splice variant c.3455+1G>A. However, the severely decreased PIEZO1 function in erythrocytes of II.2 argues against the presence of substantial amounts of functional protein produced from this allele. It would be reasonable to hypothesize that the truncated PIEZO1 variant may be dominant negative as part of a multimer subunit complex. The absence of symptoms in parent I.1, however, argue against a major dominant negative effect of this variant.

It is noteworthy that while global deletion of *Piezo1* leads to embryonic lethality in mice^8^,^16^, our patients exhibit a much milder phenotype despite severely reduced PIEZO1 functionality. It is possible that this is due to residual functional protein, which may be sufficient to maintain vascular development in the patients. It is, however, also possible that this phenotypic difference is due to species differences between mice and humans. Specific ablation of *Piezo1* in lymphatic endothelial cells in mice will yield valuable insight into the pathomechanism of this condition on the tissue development level. Furthermore, PIEZO1 functional tests in patients in this novel clinical category will help address the extent of PIEZO1 involvement in human vasculature.

The present study used erythrocytes as reporters of overall PIEZO1 function in the affected individuals, taking advantage of the PIEZO1-dependent calcium responses in these cells. We therefore propose that the novel Piezo1-activating compound Yoda1 serves as a convenient tool to assess PIEZO1 functionality using calcium imaging of red blood cells, a sample that is usually readily obtainable in a clinical setting. This method may serve as a diagnostic or screening tool in future studies.

## Methods

### Whole-exome sequencing and analysis

Whole-exome sequencing was performed on DNA extracted from whole blood using a previously described method with the following alterations: the Agilent SureSelectXT Human All Exon V4 kit (Agilent Technologies) was used^27^, and sequencing was performed on the HiSeq2500 instrument (Illumina) with 100-bp paired-end sequencing. Sequence was aligned to the human reference genome (Hg19) using Burrow–Wheeler Aligner (0.5.11)^28^; variants were called with Samtools^29^ and Genome Analysis Toolkit (v.1.6)^30^ and annotated with Annovar^31^. Variants with a quality score <10 were excluded from analysis. Variant filtering and segregation was performed with in-house developed software (https://github.com/brendanofallon/VarViewer). All genes previously associated with congenital lymphoedema (*CCBE1*, *FOXC2*, *FLT4*, *KIF11*, *GATA2*, *GJC2*, *SOX18*, *FAT4* and *PTPN14*) were specifically analysed and no rare or pathogenic variants were found in any family member despite full sequencing coverage. The reported *PIEZO1* variants were confirmed by Sanger sequencing using standard methods. Primer sequences are available upon request.

### Patient information

II.1 had an extensive clinical and diagnostic evaluation including a single-nucleotide polymorphism chromosomal microarray, lysosomal enzyme panel, screening for congenital disorders of glycosylation, sequencing of the *FOXF1* gene and a clinical gene-sequencing panel for Noonan syndrome, all of which were negative. This study was approved by the institutional review boards at the University Of Utah School Of Medicine and the Scripps Research Institute. Informed consent for DNA, RNA and functional analyses and publication of identifying photographs was obtained from study participants in line with institutional review board requirements at the time of collection.

### RT–PCR analysis

RNA was extracted from whole blood using a MagNA Pure Compact Instrument (Roche). cDNA was generated from 1 μg total RNA using the SuperScript III First Strand Synthesis kit (Life Technologies) and primed with random hexamers. RT–PCR was performed using the M13-tailed primers (5′-TGTAAAACGACGGCCAGTACTCCGCACTCATCAAGTGG-3′ and 5′-CAGGAAACAGCTATGACCTTGACGGTGCATACAAGGCT-3′) under standard conditions and analysed on a QIAxcel Electrophoresis System (Qiagen). RT–PCR amplicons were Sanger sequenced using M13 primers.

### Generation of Piezo1-deficient HEK293T (HEK-P1KO) cells

Piezo1-deficient HEK293T cells were generated using CRISPR/Cas9 nuclease genome engineering. A genomic RNA sequence 5′-TACCTGGTCGTCTGATAGGG-3′ (bases 5,997–5,978 of the hPiezo1 coding sequence, close to the 32nd transmembrane domain) was cloned into a modified version of the plasmid pSpCas9(BB)-2A-GFP^32^ (PX458, Addgene plasmid #48138, a gift from Feng Zhang) in which the coding sequence of enhanced green fluorescent protein (eGFP) was replaced by that of mCherry. HEK293T cells were transfected in six-well plates using 4 μg of plasmid combined with 10 μl of a 1-mg ml^−1^ solution of PEIMax (Polysciences) in 500 μl OptiMEM (Life Technologies). At 2 days after transfection, cells were dissociated and single fluorescent cells were sorted into individual wells of 96-well plates containing preconditioned complete media using a MoFlo Astrios cell sorter (Beckman Coulter). One clone was identified possessing a homozygous deletion of the sequence 5′-GTCCTCCC-3′ (bases 5,973–5,980), resulting in a premature truncation of Piezo1. Cells deriving from this clone exhibited no inward cation currents in response to poking, in contrast to parental HEK293T cells that often exhibited high-threshold responses.

### Cell culture and electrophysiology

HEK-P1KO cells were cultured in DMEM containing 10% heat-inactivated fetal bovine serum and 1% penicillin/streptomycin (Gibco, Life Sciences). Overexpression of all constructs was achieved with Lipofectamine 2000 according to manufacturer’s instructions (Life Sciences). Whole-cell recordings were conducted using 2–4 MΩ fire-polished borosilicate pipettes forged with a Flaming/Brown P-97 puller (Sutter Instruments). Responses were amplified with an Axopatch 200B differential amplifier, digitized using a Digidata 1,550 multi-channel digitizer, recorded and analysed using the Clampex software suite (Molecular Devices). Recordings were performed in normal extracellular media (NECM) containing (in mM): 140 NaCl, 4 KCl, 1.2 CaCl_2_, 1 MgCl_2_, 10 HEPES and 10 glucose, pH 7.4. The pipette solution (Cs4A-IC) contained (in mM): 133 CsCl, 7 KCl, 1 CaCl_2_, 5 MgCl_2_, 4 Na_2_ATP, 0.5 Na_2_GTP and 10 HEPES, pH 7.3.

### Fluorescent calcium imaging

Erythrocyte calcium imaging experiments were performed as previously described^15^. Briefly, blood was drawn and stored on ice in EGTA vacutainers. On the experimental day, we diluted whole blood 1:1,000 into NECM supplemented with 0.1% BSA and loaded with 5 μM Fluo-4 at 4 °C for at least an hour. Cells were then washed with buffer to remove excess dye and allowed to settle in a custom-build low-volume perfusion chamber, where a gravity-driven whole-chamber perfusion system was used to deliver Yoda1 and the calcium ionophore A23187 after erythrocytes loosely adhered to the uncoated glass-bottom chamber. For mechanical stimulation, we used long, tapered-tip (∼1.5–2 μm diameter) borosilicate pipettes (Sutter instruments, Novato, CA) forged with a Flaming/Brown P-97 puller (Sutter Instruments) and applied negative pressure to the cells using a High-speed Pressure Clamp device (ALA scientific, Farmingdale, NY). Imaging was performed using an Axio Observer (Zeiss) microscope fitted with a lambda DG4 fluorescent excitation source (Sutter Instruments). Images were recorded with an Orca Flash 4.0 camera (Hamamatsu Photonics) using MicroManager imaging software^33^. Image analysis was performed with FIJI^34^. HEK-P1KO cells were loaded with 2 μM Fura2-AM for 30 min at room temperature in NECM and imaged using the equipment described above.

### Materials and constructs

Yoda1 was obtained from the Genomics Institute of the Novartis Research Foundation, commercially available from Maybridge Chemical Company. A23187 was purchased from TOCRIS Bioscience. Point mutations and myc-tagged constructs were generated using Agilent Technologies XL site-directed mutagenesis kit on the hPiezo1-pIres2-EGFP template backbone according to the manufacturer’s instructions. The mutagenesis primers were as follows: hPiezo1-G2029R-fwd: 5′-aaggccagcttgcgcagcacggtcttg-3′; hPiezo1-G2029R-rev: 5′-caagaccgtgctgcgcaagctggcct-3′. To generate myc-tagged constructs, mPiezo1 and hPiezo1 sequences were aligned. Topology information obtained from previously reported myc topology screening was used to generate the human MYC13 extracellular tag construct^18^. The primers used for myc tag insertion were as follows. hPiezo1-Myc1764(myc13)-fwd: 5′-CACGTGGTGCTGCGGCGCTACGAGGAACAAAAACTTATTTCTGAAGAAGATCTGAACAAGCCCTACTTCCCGCCC-3′; hPiezo1-Myc1764(myc13)-rev: 5′-GGGCGGGAAGTAGGGCTTGTTCAGATCTTCTTCAGAAATAAGTTTTTGTTCCTCGTAGCGCCGCAGCACCACGTG-3′. All generated constructs were verified by full-length cDNA sequencing.

### Immunofluorescent staining

Live and permeabilized staining of HEK-P1KO cells was carried out as described previously^18^. Briefly, 24–48 h after transfection live labelling was carried out by incubating cells with Myc 9E11 (1:50; Santa Cruz Biotechnology) at 37 °C for 1 h. After five washes with warm medium, cells were incubated with secondary antibodies conjugated to Alexa Fluor 546 (1:200; Life Technologies) for 10–20 min at room temperature. Cells were washed five times with PBS, fixed with 2% paraformaldehyde/PBS for 20–30 min and imaged at an Olympus (Tokyo, Japan) Fluoview 500 confocal microscope by illumination with the HeNe green 543-nm laser. For permeabilized immunostaining, cells were first fixed with 4% paraformaldehyde/PBS for 10 min, washed five times with PBS, permeabilized in PBS containing 0.4% Triton X-100 and blocked with 10% normal goat serum in PBS followed by incubation with antibodies.

## Additional information

**How to cite this article:** Lukacs, V. *et al.* Impaired PIEZO1 function in patients with a novel autosomal recessive congenital lymphatic dysplasia. *Nat. Commun.* 6:8329 doi: 10.1038/ncomms9329 (2015).
